# Reconstructing schoolyards with greenery to increase schoolchildren’s physical activity and mitigate climate changes in urban areas: study protocol for a stepped-wedge trial

**DOI:** 10.1186/s12889-026-26609-9

**Published:** 2026-02-17

**Authors:** Charlotte Wilén, Viktor H. Ahlqvist, Jairo Hidalgo Migueles, Pablo Campos-Garzón, Micael Dahlén, Kyriaki Kosidou, Karin Rådholm, Pontus Henriksson, Daniel Berglind

**Affiliations:** 1 Department of Global Public Health, Karolinska Institutet, Stockholm, Sweden; 2 Department of Biomedicine, Aarhus University, Aarhus, Denmark; 3 Institute of Environmental Medicine, Karolinska Institutet, Stockholm, Sweden; 4 PROFITH “PROmoting FITness and Health through physical activity” Research Group, Department of Physical Education and Sports, Faculty of Sport Sciences, University of Granada, Granada, Spain; 5 Center for Wellbeing, Welfare and Happiness, Stockholm School of Economics, Stockholm, Sweden; 6 Centre for Epidemiology and Community Medicine (CES), Stockholm, Sweden; 7 Primary Health Care Centre Kärna and, Department of Health, Medicine and Caring Sciences, Linköping University, Linköping, Sweden; 8 Department of Health, Medicine and Caring Sciences, Linköping University, Linköping, Sweden

**Keywords:** Children’s physical activity, Accelerometry, Climate change, Schoolyard reconstruction

## Abstract

**Background:**

The benefits of physical activity are well-documented, and healthy habits established in childhood often continue into adulthood. Recent research has shown that schoolyards provide a valuable platform for children to be physically active, with greener spaces in particular enhancing both physical and mental well-being. The City of Stockholm has formally decided to reconstruct 20 schoolyards, incorporating more play areas and greenery. This study will evaluate the impact of these reconstructions, aiming to increase physical activity levels among schoolchildren across all socioeconomic groups, while also contributing to climate change mitigation in urban environments.

**Methods:**

This study will utilize a stepped-wedge design, where each school undergoing schoolyard reconstruction will serve as both a control and intervention site. Over four years, from 2024 to 2027, five schools will have their schoolyards reconstructed each summer. Control data will be collected in the spring prior to the reconstruction, with follow-up data collected in the spring after the reconstruction. We aim to recruit 3 600 children aged 6 to 12 years. The primary outcome will be changes in physical activity, measured via accelerometers. Secondary outcomes will include changes in musculoskeletal fitness, perceptions of the schoolyard, and environmental impact. Given the 24-hour constraint of daily time, movement behaviors (e.g., MVPA, LPA, SB, and sleep) will be treated as compositional data. Log-ratio transformation will be applied and introduced as outcomes in general linear mixed models, with schools treated as random effects.

**Discussion:**

This large-scale study has the potential to set new guidelines for physical health policies in schools across the City of Stockholm, potentially influencing the well-being of an even greater number of children. Additionally, the study could provide valuable insights into strategies for mitigating climate change through urban design, offering a model for sustainable school environments that promote both health and environmental resilience.

**Trial registration:**

The trial has been registered on ClinicalTrials.gov the 19th of May 2023, with the reference number NCT05865782.

**Supplementary Information:**

The online version contains supplementary material available at 10.1186/s12889-026-26609-9.

## Background

The health benefits of a physically active lifestyle during childhood are well-documented and include for example improved cardiometabolic health, mental health, sleep and positive effects on weight maintenance [1].

Establishing healthy physical activity behaviors in childhood may track into adulthood [2]. Moreover, traditional cardiovascular risk factors such as childhood levels of body mass index (BMI) and systolic blood pressure are associated with the development of incident adult cardiovascular events [3]. As such, primary prevention aiming at establishing healthy physical activity behaviors in young childhood may have long-lasting effects on chronic disease risk in adulthood.

In spite of the growing awareness of the health benefits from physical activity, less than half of children and adolescence worldwide reach the guidelines for physical activity [4] – and the same holds true for Sweden. More than half of Swedish school children do not meet the current guidelines, with girls being less active than boys and children having a lower socioeconomic status being less active than their socioeconomically privileged peers [5]. Since elementary school participation is mandatory in Sweden, the school environment is a fundamental arena for establishing healthy physical activity behaviors on a population level, independent of socioeconomic factors.

A Cochrane review from 2021, including 89 studies, showed that school-based PA interventions can have a small effect on children’s MVPA levels during school-hours and that school recess is a key setting that can provide opportunities for children to spent time outdoors and play and be physically active [6]. Another review showed that school recess can contribute between 5% and 40% of children’s recommended daily PA levels and that the least physically active children accumulate most of their daily PA during recess [7]. Systematic review data from intervention studies (*n* = 13) show moderate evidence for an effect on children’s PA levels from the provision of play equipment and limited evidence for an effect of decreasing playground density [8]. Moreover, a Danish intervention study from 2020 showed that a schoolyard reconstruction (e.g., implementation of new play features in the schoolyard such as climbing walls, balance-bars, skating areas, trampolines, hills, ball game facilities etc.) may increase children’s time spent outdoors and device-measured PA levels [9]. A systematic review from 2021, including 6 intervention studies, show that schoolyard greening has an effect both on physical activity and socioemotional health outcomes for school children [10]. Taken together, these data support the importance of the schoolyard as an important setting to increase children’s PA levels.

Beyond the immediate health benefits, increasing greenery in urban areas also addresses broader global concerns. Due to human activity, health and environmental risks due to climate effects in urban areas are expected to increase, with subsequent profound negative health consequences [11]. Research has shown that green areas are a relatively simple and cost-effective strategy for climate adaptation such as air temperature regulation and pollution reduction [12]. Indeed, the current evidence show protective effects of greenspace exposure on aspects related to chronic disease risk, for example physical activity, sleep quality, mental health and immunity function [13, 14]. Moreover, planting trees with heavy foliage to increase shade of schoolyards may be an effective strategy to reduce schoolchildren’s exposure to excessive sun exposure and the development of melanoma [15]. Consequently, increasing green area coverage in cities, via schoolyard reconstructions with greenery, is a promising and novel strategy to mitigate climate changes in urban environments and improve short- and long-term population health.

A formal decision in the Stockholm City Hall was taken to reconstruct 20 Stockholm public schoolyards with increased greenery during the years 2024–2027, (five schoolyard reconstructions per year). The proposed project entails an evaluation of the 20 schoolyard renovations. This is a unique co-creation project between Swedish Cancer Society, Birthe & Per Arwidsson’s Foundation, the City of Stockholm and Karolinska Institutet with the ambitious aim to increase school children’s physical activity levels across all socioeconomic groups and mitigate climate changes in urban environments.

## Objectives

### Aims

The primary aim of this project is to examine the effectiveness of schoolyard reconstruction on schoolchildren’s physical activity levels. The secondary aims include examining the effectiveness of schoolyard reconstructions on children’s i) musculoskeletal fitness ii) psychosocial functioning iii) sleep iv) perception of the schoolyard quality v) screentime, as well as on vi) incidents in the schoolyard, vii) the cost-effectiveness of schoolyard reconstruction and, viii) the environmental impact of schoolyard reconstruction.

### Hypothesis

Our main hypothesis is that schoolyard reconstructions will increase physical activity levels in school children and that reconstruction components such as greater greening and the provision of play equipment will have the strongest association with children’s changes in health outcomes. We further hypothesize that the reconstructions will have a positive impact on schoolyard quality and on the urban environment.

## Materials and methods

### Study design and setting

**Fig. 1:**
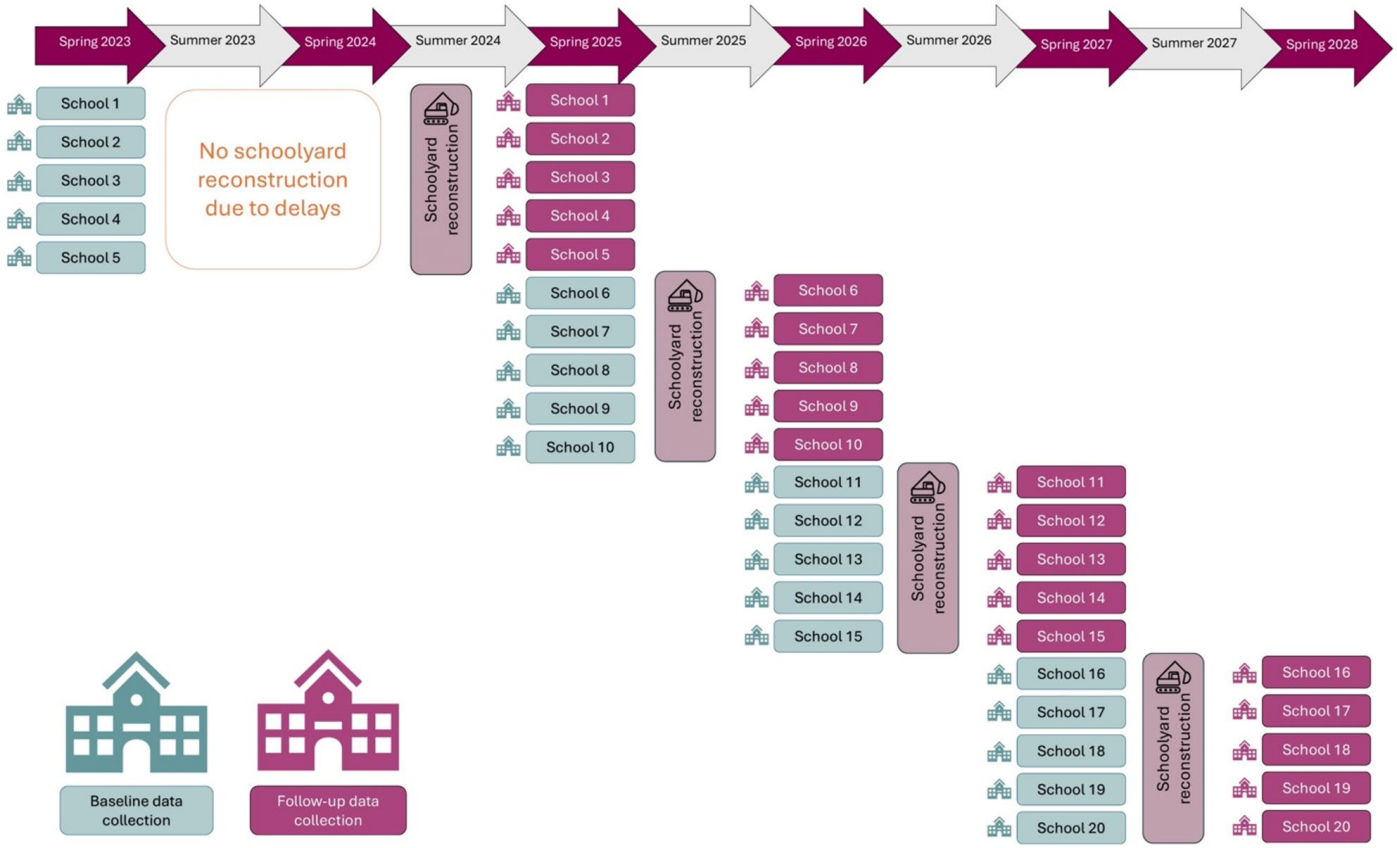
Time frame and study design of the project

The current study will be designed as a stepped-wedge intervention where schools undertaking schoolyard reconstruction will act as both control and intervention schools [16]. A cross-sectional sample of children in 1st to 6th grade will be measured annually in all participating schools. Children will not be tracked longitudinally; therefore, the pre- and post-intervention samples will not necessarily include the same individuals. As a result, we avoid the issue of individuals being older at follow-up. Each year, five schools located in Stockholm County will receive the intervention—schoolyard reconstruction—during the summer breaks of 2024, 2025, 2026, and 2027 (see Fig. 1). The schools will act as control sites during the spring before the reconstruction and as intervention sites in the spring after the reconstruction. This design allows us to adjust for within-school characteristics, as each school serves as its own control. Additionally, by scheduling data collection at similar time points before and after the intervention within each school, we seek to minimize the influence of seasonal variation, which may affect outcomes such as children’s physical activity levels [17]. Furthermore, a stepped-wedge study design [16], is susceptible to year-to-year variation, and will therefore be confounded by time [18]. To address this, the five schools receiving their baseline measurements during the same spring period in which the intervention schools receive their follow-up measurements will be included in the analysis, allowing us to adjust for temporal variation across years. This report follows the SPIRIT reporting guidelines [19], with a checklist provided in additional file 1.

### Sample size

We have calculated the sample size to be able to detect an effect for treating each intervention-control schoolyard reconstruction as an independent unit analyzed separately. Power calculations were based on MVPA as the primary outcome, with a target power of 0.90. A five minute change in MVPA has been suggested in previous meta-analysis to be a reasonable target a of clinical importance [20]. Under the assumption of a standard deviation of 20 min [5], a minimum of 70 children per school at each pre‑ and post‑measurement is required. Although the study will include 20 schools, this sample size ensures sufficient power for analyses conducted at earlier stages of the stepped‑wedge design, when fewer schools are available.

### Study population and recruitment

We have chosen to include children in 1st to 6th grade since some schools have few classes; thus, we need to include children from different ages to meet the required sample size. Based on our recent study, including > 3 500 preschoolers with accelerometer-measured physical activity [21], approximately 20% total non-participation is expected throughout the study period. Consequently, we aim to recruit a sample of 90 children from each participating school at baseline and follow-up, i.e., in total 180 children for each participating school. In total, the study population will include 3 600 children from 20 schools undertaking schoolyard reconstructions over four years (180 × 20 = 3 600).

### Inclusion/exclusion criteria

Schools undergoing schoolyard reconstruction have been chosen by the Stockholm education administration depending on their need for schoolyard renewal and geographical location to represent areas in Stockholm with different socioeconomic circumstances. The children included in the study are enrolled in the participating school, 6–12 years of age at the time of study start, in 1st -6th grade and participating with the consent of their guardians. All children that fulfill the criteria will be invited to participate. Classes for students with disabilities will be included in some schools, with no restrictions based on type or severity of disability.

### Intervention description

Since 2015, the Education Committee of Stockholm has been undertaking the reconstruction of schoolyards to address the need for evaluating their quality and exploring innovative approaches for schoolyard development. In the 2023 and 2024 City Council budget, a special initiative was allocated to the Education Committee, tasking them with developing a concept and strategy for greener, more sustainable schoolyards. After the decision was made on which schools would undergo reconstruction, Karolinska Institutet was invited to evaluate the process. Thus, Karolinska Institutet is not involved in the selection of schools but is solely responsible for evaluating the reconstruction process.

The schoolyard reconstruction process will use a participatory bottom-up co-creation development approach involving students, school personnel and researchers and be evidence-based, using prior knowledge on schoolyard features that are associated with children’s PA levels during school-hours [8]. Additionally, the schoolyard reconstruction intervention process will be discussed and acknowledged among all co-creators to increase the engagement, buy-in, feasibility, relevance and sustainability of the schoolyard reconstruction intervention as recommended by a previous meta-analysis on the effectiveness in co-creation of research [22].

Workshops will be held with children, school personnel (e.g., principal, vice-principal etc.), landscape architects and researchers, at each school before schoolyard reconstruction initiation. The workshops will process the design and dimension of what components the schoolyard renewal should encompass, reflecting local challenges and needs. At some of the schools the schoolyard renewal will take place in the existing schoolyard, whereas other schools will expand their outdoor area by including adjacent spaces (e.g., woodland area and parking ground). However, the most essential change will encompass implementation of new play features/equipment in the schoolyard such as climbing walls, balance-bars, trampolines, hills, and ball game facilities, that will be applied at most of the schools.

### Outcomes and measures

To assess the outcomes, various measurements have been used and can be seen in additional file 2. Questionnaires are distributed via email to guardians, who are asked to respond to questions about both themselves and their children. The questionnaire about schoolyard quality is answered by the child. In the first year of data collection, all questionnaires were available only in Swedish, but were thereafter translated to English, to extend the language options (additional file 3, 4 and the Strengths and Difficulties Questionnaire [23]). The questionnaire answered by the contact person on the school was only available in Swedish and has been translated to English in additional file 5. Questionnaires in Swedish can be shared upon request. Moreover, the characteristics of each schoolyard will be documented before and after reconstruction to enable the assessment of how specific features influence the observed outcomes.

### Change in physical activity levels (primary outcome)

Physical activity will be measured objectively by GT3X+ accelerometers and will be categorized as moderate to vigorous physical activity (MVPA) light physical activity (LPA) sedentary time (ST) and steps with the primarily focus on MVPA. The children will be asked which one is their dominant hand, and they will be instructed to wear the accelerometer on their non-dominant hand for 24 h for 7 consecutive days, except during water activities. The sampling frequency will be at 30 hz, and an epoch length of 5 s will be used as recommended for children and adolescents [24]. The software Actilife will be used to download the data from the accelerometers and the data will then be analyzed using the open-source R package GGIR [25].

To assess the impact of the schoolyard reconstruction on children’s physical activity, we will categorize their activity levels during school hours and outside of school hours. Since many children in this age group attend afterschool care, which takes place outside of regular school hours but still occurs on school premises, guardians will be asked to indicate whether their child attended afterschool care and the time they finished.

### Change in musculoskeletal fitness in children (secondary outcome)

Musculoskeletal fitness will be measured as hand grip strength using an analogue dynamometer (TKK 5825, Grip-A, Takei, Tokyo, Japan). Two measurements will be taken on each hand and will be rounded to nearest 0.5 kg.

### Change in psychosocial functioning in children (secondary outcome)

Psychosocial functioning in children will be assessed by a parental proxy report of the Strengths and Difficulties Questionnaire [23]. A total score will be generated by adding the scores from all scales, except the prosocial scale, resulting in a range between 0 and 40. Higher score means worse outcome. Further, happiness will be assessed using a single-item Face Scale [26], consisting of seven smiley faces, answered by the children.

### Change in sleep in children (secondary outcome)

Children’s sleep will be measured by the GT3X+ accelerometers. Algorithms integrated in the GGIR package will distinguish sleet timing, duration, and continuity [27].

### Change in children’s perception of the schoolyard quality (secondary outcome)

A questionnaire with 7 items, previously used to evaluate schoolyard reconstructions [28], will be used to assess children’s perception of the schoolyard quality.

### Children’s screentime (secondary outcome)

Guardians will report their child’s screen time by answering questions about the average duration of screen use (in minutes) on weekdays and weekends, respectively.

### Children’s opportunities to be physically active outside of school (process outcome)

Parents will provide information about their child’s mode of transportation to and from school, if the child participate in organized sports, as well as their own physical activity habits.

### Schoolyard reconstruction costs (secondary outcome)

Detailed budget costs for each schoolyard reconstruction, e.g., play equipment, construction and maintenance costs will be collected for each separate schoolyard reconstruction and further used for cost-effectiveness analysis (CEA) and a cost-benefit analysis (CBA). In the CEA, we will compare the costs of the intervention to the health outcomes achieved. The CBA will go further to include monetary valuation of environmental benefits.

### Environmental impact of schoolyard reconstruction (secondary outcome)

To assess the environmental effects of the schoolyard reconstructions, we will analyze changes in tree coverage and surface reflectivity (albedo, Δα) using detailed architectural drawings. Additionally, we will obtain temperature and PM2.5 (particulate matter) data from the Environment and Health Department to evaluate changes in air quality and thermal environment associated with the increased greenery.

### Incidents in the schoolyard (secondary outcome)

Number of incidents (e.g., violence, conflicts, sun burns etc.) from before to after schoolyard reconstruction will be collected from the schools.

### Demographic variables

Socioeconomic status will be measured using parental education, asked about in one of the questionnaires. Age and sex will be determined from the social security number [29], collected through the consent forms. This number includes the individual’s date of birth, and the second-to-last digit indicates the individual’s sex. If the last digits of the social security number are missing, sex will instead be determined based on questionnaire data. Parents will also be asked to report their occupation, country of birth, and the height and weight of both themselves and their children.

### Statistical analysis

Descriptive characteristics of the participants will be summarized using summary statistics.

The unit of analysis will be children nested within schools using a random effect. Generalized linear mixed models will be used to analyze changes in time spent at different physical activity intensities and secondary outcomes, comparing measurements taken before and after the schoolyard reconstruction. When appropriate, compositional data analysis will be employed to examine all daily activity—physical activity, sedentary time, and sleep—behaviors collectively [30]. This approach treats these behaviors as interdependent parts of a finite whole (i.e., the 24-hour day), acknowledging that an increase in time spent in one behavior necessarily results in a decrease in one or more of the others. Rather than focusing on isolated changes (e.g., an increase in MVPA alone), compositional data analysis allows for the analysis of shifts in the balance among all daily behaviors. The compositional outcome will be expressed through isometric log-ratio transformations, which recognize that each behavior’s duration is meaningful only in relation to the others. For easier interpretation, isometric log-ratios will be back-transformed into minutes to illustrate how the intervention influences the distribution of time across movement behaviors.

Physical activity will be further analyzed by distinguishing between time spent during school hours, at recess, and outside of school hours. As described in the Study Design and Setting section, temporal variation across calendar years will be controlled by including a period-of-measure indicator together with the intervention indicator (pre- or post-intervention measure).

### Result dissemination

The research team will have a key role in communicating the results from the study to stakeholders and all end-users (the City of Stockholm, school management, teachers, children/guardians). We shall disseminate our results to relevant authorities and decision-makers, including the Swedish Association of Local Authorities and Regions and policy makers in Swedish municipalities. Results will also be presented at several national and international scientific conferences and in Swedish lay media. The main results will further be presented in the form of a project website.

### Time plan of the project

This project entails an evaluation of five schoolyard reconstructions per year, in total 20 schoolyard reconstructions over four years. The reconstruction process is estimated to take between three to four months during the summer break. The schoolyard reconstructions were originally scheduled for 2023–2026. However, due to a recent increase in reconstruction costs, the projects planned for 2023 was postponed to 2024. Consequently, the follow-up data collection for the first five schools will now take place the spring of 2025 as shown in Fig. 1.

The recruitment of participants began in May 2023 and follow-up measurements for the first group of schools will take place in spring 2025. The remaining schools are expected to follow to the time plan, with recruitment expected to be completed by spring 2028 (see Fig. 1). The trial has been registered on ClinicalTrials.gov the 19th of May 2023 with the reference number NCT05865782. This is the first version of the protocol. Any changes to the protocol will be communicated to the trial registry.

## Discussion

This study aims to evaluate the impact of schoolyard reconstructions that incorporate greenery on children’s health and environmental outcomes in urban settings. Additionally, we will assess the cost-effectiveness of these redesigned schoolyards by comparing their implementation costs with the resulting health benefits for children. As a large-scale co-creation initiative, this project will contribute valuable evidence to the scientific literature and inform the development of healthier, greener schoolyards in urban environments.

There are several strengths in this study. The first lies in the study’s design; having schools serve as their own control naturally controls for confounding factors, such as socioeconomic status and school policies, thereby helping to isolate the effect of the intervention. Additionally, this approach ensures that all participating schools receive the intervention, which enhances the study’s ethical favorability. Furthermore, fewer schools are required compared to a design that involves separate control groups. A second key strength is that children’s physical activity will be measured objectively using accelerometers, providing a more accurate estimate than self-reported measurements. Third, geographical location and the representation of different socioeconomic groups in Stockholm were considered when selecting the participating schools. This approach will contribute to a more diverse study population and enable the examination of children’s health based on socioeconomic characteristics.

The study does have some limitations. Schools are selected based on their geographical location to represent different socioeconomic groups in Stockholm and their need for schoolyard reconstruction. As a result, the schools are not randomly selected, which can introduce potential biases. By excluding schools that may not require reconstruction as urgently, the study population may differ from the broader target population, potentially affecting the generalizability of the findings. There is also a limitation in the study design; schools serve as their own controls rather than having parallel and separate control groups, which leaves the design more susceptible to seasonal variation, which must be accounted for in the statistical analysis. Additionally, certain measurements, such as the height and weight of the children and guardians, are obtained through self-reported questionnaires rather than objective methods. This may lead to inaccuracies in the data, as self-reported measures are often less reliable.

This study will provide valuable insights into the impact of schoolyard reconstruction on climate mitigation and children’s physical activity. The findings can inform new guidelines and offer evidence to guide future schoolyard renovations, promoting increased physical activity and enhanced climate mitigation in more urban schools.

## Supplementary Information

Supplementary Material 1.

Supplementary Material 2.

Supplementary Material 3.

Supplementary Material 4.

Supplementary Material 5.

Supplementary Material 6.

## Data Availability

The datasets accrued within the study is stored in a “de-identified” (pseudo anonymous) manner in a password-protected area of the server at Karolinska Institutet and will be available to investigators affiliated with Karolinska Institutet. We aim for public access of statistical code. However, the datasets cannot be available for the public due to Swedish legislations and restrictions in ethical approval. All data within the framework of the current study will be unidentified and presented as aggregated statistical measures, such as means, relative risks, etc. The key variable and its link to personal identifiers will be kept separated from the measurement data. Sensitive information will be handled without personal identifiers and data will be presented as aggregated statistical measures without the possibility for personal identification.

## Abbreviations

MVPA: Moderate to vigorous physical activity
LPA: Light physical activity
ST: Sedentary time

## References

[1] Chaput JP, Willumsen J, Bull F, Chou R, Ekelund U, Firth J, et al. 2020 WHO guidelines on physical activity and sedentary behaviour for children and adolescents aged 5–17 years: summary of the evidence. Int J Behav Nutr Phys Act. 2020;17(1):141. 33239009 10.1186/s12966-020-01037-z PMC7691077

[2] Garcia-Hermoso A, Izquierdo M, Ramirez-Velez R. Tracking of physical fitness levels from childhood and adolescence to adulthood: a systematic review and meta-analysis. Transl Pediatr. 2022;11(4):474–86. 35558968 10.21037/tp-21-507 PMC9085944

[3] Jacobs DR Jr., Woo JG, Sinaiko AR, Daniels SR, Ikonen J, Juonala M, et al. Childhood cardiovascular risk factors and adult cardiovascular events. N Engl J Med. 2022;386(20):1877–88. 35373933 10.1056/NEJMoa2109191 PMC9563825

[4] Aubert S, Barnes JD, Abdeta C, Abi Nader P, Adeniyi AF, Aguilar-Farias N, et al. Global matrix 3.0 physical activity report card grades for children and youth: results and analysis from 49 countries. J Phys Act Health. 2018;15(S2):S251–73. 30475137 10.1123/jpah.2018-0472

[5] Nyberg G, Kjellenberg K, Froberg A, Lindroos AK. A National survey showed low levels of physical activity in a representative sample of Swedish adolescents. Acta Paediatr. 2020;109(11):2342–53. 32266736 10.1111/apa.15251

[6] Neil-Sztramko SE, Caldwell H, Dobbins M. School-based physical activity programs for promoting physical activity and fitness in children and adolescents aged 6 to 18. Cochrane Database Syst Rev. 2021;9:CD007651. 34555181 10.1002/14651858.CD007651.pub3 PMC8459921

[7] Ridgers ND, Stratton G, Fairclough SJ. Physical activity levels of children during school playtime. Sports Med. 2006;36(4):359–71. 16573359 10.2165/00007256-200636040-00005

[8] Broekhuizen K, Scholten AM, de Vries SI. The value of (pre)school playgrounds for children’s physical activity level: a systematic review. Int J Behav Nutr Phys Act. 2014;11:59. 24885611 10.1186/1479-5868-11-59 PMC4031969

[9] Skau Pawlowski C, Bondo Andersen H, Schipperijn J. Difference in outdoor time and physical activity during recess after schoolyard renewal for the Least-Active children. J Phys Act Health. 2020;17(10):968–76. 32858525 10.1123/jpah.2019-0270

[10] Bikomeye JC, Balza J, Beyer KM. The impact of schoolyard greening on children’s physical activity and socioemotional health: A systematic review of experimental studies. Int J Environ Res Public Health. 2021;18(2):535. 33561082 10.3390/ijerph18020535 PMC7827958

[11] Romanello M, Di Napoli C, Drummond P, Green C, Kennard H, Lampard P, et al. The 2022 report of the lancet countdown on health and climate change: health at the mercy of fossil fuels. Lancet. 2022;400(10363):1619–54. 36306815 10.1016/S0140-6736(22)01540-9 PMC7616806

[12] Quaranta E, Dorati C, Pistocchi A. Water, energy and climate benefits of urban greening throughout Europe under different Climatic scenarios. Sci Rep. 2021;11(1):12163. 34108503 10.1038/s41598-021-88141-7 PMC8190137

[13] Porcherie M, Linn N, Le Gall AR, Thomas MF, Faure E, Rican S, et al. Relationship between urban green spaces and cancer: A scoping review. Int J Env Res Pub He. 2021;18(4):1751. 10.3390/ijerph18041751 PMC7916941 33670207

[14] Yang BY, Zhao T, Hu LX, Browning M, Heinrich J, Dharmage SC, et al. Greenspace and human health: an umbrella review. Innov (Camb). 2021;2(4):100164. 10.1016/j.xinn.2021.100164 PMC8479545 34622241

[15] Reyes-Marcelino G, Wang R, Gultekin S, Humphreys L, Smit AK, Sharman AR, et al. School-based interventions to improve sun-safe knowledge, attitudes and behaviors in childhood and adolescence: A systematic review. Prev Med. 2021;146:106459. 33609617 10.1016/j.ypmed.2021.106459

[16] Copas AJ, Lewis JJ, Thompson JA, Davey C, Baio G, Hargreaves JR. Designing a stepped wedge trial: three main designs, carry-over effects and randomisation approaches. Trials. 2015;16:352. 26279154 10.1186/s13063-015-0842-7 PMC4538756

[17] Rich C, Griffiths LJ, Dezateux C. Seasonal variation in accelerometer-determined sedentary behaviour and physical activity in children: a review. Int J Behav Nutr Phys Act. 2012;9:49. 22546178 10.1186/1479-5868-9-49 PMC3511197

[18] Hemming K, Haines TP, Chilton PJ, Girling AJ, Lilford RJ. The stepped wedge cluster randomised trial: rationale, design, analysis, and reporting. BMJ. 2015;350:h391. 25662947 10.1136/bmj.h391

[19] Hrobjartsson A, Boutron I, Hopewell S, Moher D, Schulz KF, Collins GS, et al. SPIRIT 2025 explanation and elaboration: updated guideline for protocols of randomised trials. Bmj-British Med J. 2025;389:e081660. 10.1136/bmj-2024-081660 PMC12128891 40294956

[20] Metcalf B, Henley W, Wilkin T. Effectiveness of intervention on physical activity of children: systematic review and meta-analysis of controlled trials with objectively measured outcomes (EarlyBird 54). BMJ. 2012;345:e5888. 23044984 10.1136/bmj.e5888

[21] Chen C, Ahlqvist VH, Henriksson P, Migueles JH, Christiansen F, Galanti MR, et al. Increasing children’s physical activity by policy (CAP) in preschools within the Stockholm region: study protocol for a pragmatic cluster-randomized controlled trial. Trials. 2022;23(1):577. 35854370 10.1186/s13063-022-06513-4 PMC9295109

[22] Halvorsrud K, Kucharska J, Adlington K, Rudell K, Brown Hajdukova E, Nazroo J, et al. Identifying evidence of effectiveness in the co-creation of research: a systematic review and meta-analysis of the international healthcare literature. J Public Health (Oxf). 2021;43(1):197–208. 31608396 10.1093/pubmed/fdz126 PMC8042368

[23] Goodman R. The strengths and difficulties questionnaire: a research note. J Child Psychol Psychiatry. 1997;38(5):581–6. 9255702 10.1111/j.1469-7610.1997.tb01545.x

[24] Migueles JH, Cadenas-Sanchez C, Ekelund U, Delisle Nystrom C, Mora-Gonzalez J, Lof M, et al. Accelerometer data collection and processing criteria to assess physical activity and other outcomes: A systematic review and practical considerations. Sports Med. 2017;47(9):1821–45. 28303543 10.1007/s40279-017-0716-0 PMC6231536

[25] Migueles JH, Rowlands AV, Huber F, Sabia S, van Hees VT. GGIR: A research Community–Driven open source R package for generating physical activity and sleep outcomes from Multi-Day Raw accelerometer data. J Meas Phys Behav. 2019;2(3):188–96.

[26] Holder MD, Klassen A. Temperament and happiness in children. J Happiness Stud. 2010;11(4):419–39.

[27] van Hees VT, Sabia S, Jones SE, Wood AR, Anderson KN, Kivimaki M, et al. Estimating sleep parameters using an accelerometer without sleep diary. Sci Rep. 2018;8(1):12975. 30154500 10.1038/s41598-018-31266-z PMC6113241

[28] Christiansen LB, Toftager M, Pawlowski CS, Andersen HB, Ersbøll AK, Troelsen J. Schoolyard upgrade in a randomized controlled study design—How are school interventions associated with adolescents’ perception of opportunities and recess physical activity. Health Educ Res. 2017;32(1):58–68. 28115424 10.1093/her/cyw058 PMC5914349

[29] Ludvigsson JF, Otterblad-Olausson P, Pettersson BU, Ekbom A. The Swedish personal identity number: possibilities and pitfalls in healthcare and medical research. Eur J Epidemiol. 2009;24(11):659–67. 19504049 10.1007/s10654-009-9350-y PMC2773709

[30] Dumuid D, Stanford TE, Martin-Fernandez JA, Pedisic Z, Maher CA, Lewis LK, et al. Compositional data analysis for physical activity, sedentary time and sleep research. Stat Methods Med Res. 2018;27(12):3726–38. 28555522 10.1177/0962280217710835

