# Supplementary material for: Reconstructing schoolyards with greenery to increase schoolchildren’s physical activity and mitigate climate changes in urban areas: study protocol for a stepped-wedge trial
Source: BMC Public Health. 2026 Feb 17;26:708. doi: 10.1186/s12889-026-26609-9 (PMC12930922; doi:10.1186/s12889-026-26609-9)
Supplement: Supplementary file 2 — Supplementary Material 2. [file 12889_2026_26609_MOESM2_ESM.docx]

| Additional file 2. Instruments and outcomes assessed. | | | | |
| --- | --- | --- | --- | --- |
| **Questionnaires** |  | **Outcomes assessed** |  | **Answered by** |
| Guardian's personal information |  | Guardian’s education, employment, birth country, physical activity, sleep, leisure activities together with child and individually, distance and mode of communication to school, children's participation in organized sports. Weight and height of guardian and child. Children’s involvement in afterschool care. Children’s screentime. |  | Guardians |
|  |  |  |  |  |
| Perceptions of the schoolyard |  | Space for play, socializing, and schoolyard equipment. Children’s happiness. |  | Children |
|  |  |  |  |  |
| The Strengths and Difficulties Questionnaire |  | Phychosocial functioning of children |  | Guardians |
|  |  |  |  |  |
| School policies |  | School policies to promote children's physical activity and healthy habits |  | The contact person on the schools |
|  |  |  |  |  |
| **Instruments** |  |  |  |  |
| Accelerometer GT3X+ |  | Physical activity levels, sedentary time and sleep of children |  |  |
|  |  |  |  |  |
| TKK 5825, Grip-A, Takei, Tokyo, Japan |  | Musculoskeletal fitness of children |  |  |

|  |  |  |  |  |
| --- | --- | --- | --- | --- |
