# Supplementary material for: Reconstructing schoolyards with greenery to increase schoolchildren’s physical activity and mitigate climate changes in urban areas: study protocol for a stepped-wedge trial
Source: BMC Public Health. 2026 Feb 17;26:708. doi: 10.1186/s12889-026-26609-9 (PMC12930922; doi:10.1186/s12889-026-26609-9)
Supplement: Supplementary file 3 — Supplementary Material 3. [file 12889_2026_26609_MOESM3_ESM.pdf]

Caregiver's personal information

Please complete the survey below.

Please note that the survey consists of a total of three questionnaires. The first two should be answered by the guardians, and the third and final one should be answered by the child.

You have the option to save your responses and return to complete the questionnaire if necessary. You can do this at the bottom of the survey.

You will automatically be redirected to the next, and final, questionnaire when you confirm that you are finished with this one.

Thank you!

Basic information on parents: Renovation of schoolyards: Effects of schoolyard renovations on school children's physical activity and health

In this quesonnaire you will be answering a few quesons about yourself. All your personal data will be handled in accordance with EU's General Data Protection Regulation (GDPR). Data analysis and data presentation will be on group level. This means that the result of the study cannot be used to identify a specific preschool, or a specific child or parent.

We appreciate your participation!

Parent's name: ______________________________
(First and last name)

Gender:
- ◯ Man
- ◯ Woman
- ◯ Other

Child's name: ______________________________
(First and last name)

Child's school: ______________________________

Child's class: ______________________________
(Example: 5B)

What is your highest level of completed education?
- ◯ Elementary school, folk school, secondary modern school or similar
- ◯ 2 years of upper secondary school or professional school
- ◯ 3-4 years of upper secondary school
- ◯ Adult education college or similar
- ◯ University, less than 3 years
- ◯ University, more than 3 years

What is your current employment status? You may choose more than one alternative	☐ Employed, please specify % of full time employment (in a text box below the options, which appears when you check the box)<br>☐ Self-employed<br>☐ On leave or parental leave<br>☐ Studying or doing an internship<br>☐ Part of a labour market policy program<br>☐ Looking for employment<br>☐ Retired<br>☐ On disability ("Sjuk- eller aktivitetsersättning")<br>☐ On sick leave<br>☐ Homemaker<br>☐ Other, please specify (in a text box that appears when you check the box)
Employed, working percentage of full-time:	___________________________________<br>(Example: 100%, 50%, etc.)
Here you specify which profession or job pertains to "other":	___________________________________
What is, or used to be, your main profession?<br><br>If you are currently not working, please describe what used to be your main profession or work. If you have several<br>jobs, please fill in the one that is your primary job. Please try to write as detailed as possible. For example, write<br>primary school teacher instead of teacher, bus driver instead of driver, etc.	___________________________________
What is your country of birth?	◯ Sweden<br>◯ Other (in a text box that appears below the question when you check the box)
Please specify which country:	___________________________________
The following questions focus on your leisure time, both together with your child and by yourself.	
On a regular weekend, I am outside with my child:	◯ About 1 hour/day<br>◯ 1-2 hours/day<br>◯ 2-3 hours/day<br>◯ More than 3 hours/day<br>◯ We usually do not go outside on weekends
When I am outside with my child on weekends:	◯ I usually watch while my child plays<br>◯ I usually participate in the play with my child<br>◯ We usually do not go outside on weekends<br>(Please select the answer that fits best)
When I am outside with my child, we usually	☐ Go to a park/green area<br>☐ Go out into nature<br>☐ We usually do not go outside on weekends<br>☐ Other (in a text box below the question that appears when you check the box)<br>(You may choose more than one answer)

Other, please describe what you do: __________________________________

In the questions below, we make a distinction between physical activity at a moderate level and exercising at a more intense level. You can think of daily physical activity as something that you do every day; for instance walk or bike, clean the home, take the stairs instead of the elevator, have a physically demanding job. When you exercise at an intense level your heart rate increases above moderate, and it usually involves showering after

Please select the alternative that best describes your level of daily physical activity:

- ◯ My level of daily exercise is much too low
- ◯ A little more daily exercise would be good
- ◯ My level of daily exercise is sufficient

How much time do you spend in an average week on exercise that makes you breathless, e.g., running or strength training?

- ◯ 0 minutes / no time
- ◯ Less than 30 minutes
- ◯ 30-60 minutes (0.5-1 hour)
- ◯ 60-90 minutes (1-1.5 hours)
- ◯ 90-150 minutes (1.5-2.5 hours)
- ◯ 150-300 minutes (2.5-5 hours)
- ◯ More than 300 minutes (5 hours)

How much time do you sit during a typical day, excluding sleep?

- ◯ Almost the entire day
- ◯ 13-15 hours
- ◯ 10-12 hours
- ◯ 7-9 hours
- ◯ 4-6 hours
- ◯ 1-3 hours
- ◯ Never

How far is it to school? __________________________________
(Answer in kilometers, e.g., 1.6 km)

How tall is your child?

- ◯ Answer in whole centimeters (in a text box that appears when you check the box)
- ◯ Don't know/Prefer not to say

Height (cm): __________________________________
(Answer in whole cm)

How much does your child weigh?

- ◯ Answer in whole kilograms (in a text box that appears when you check the box)
- ◯ Don't know/Prefer not to say

Weight (kg): __________________________________
(Answer in whole kg)

How tall are you?

- ◯ Answer in whole centimeters (in a text box that appears when you check the box)
- ◯ Don't know/Prefer not to say

Height (cm): __________________________________
(Answer in whole cm)

How much do you weigh?

- ◯ Answer in whole kilograms (in a text box that appears when you check the box)
- ◯ Don't know/Prefer not to say

Weight (kg)

____
(Answer in whole kilograms)

Does your child participate in any form of organized sports?

- ◯ No
- ◯ Yes, 1-2 hours/week
- ◯ Yes, 2-3 hours/week
- ◯ Yes, 3-4 hours/week
- ◯ Yes, more than 5 hours/week

How do you usually get to school?

- ◯ By car
- ◯ Public transport (bus, train, subway, etc.)
- ◯ Walk
- ◯ Bicycle/scooter
- ◯ Other (in a text box that appears below the question when you check the box)

Specify what "other" means:

____

Does the child attend afterschool care?

- ◯ Yes
- ◯ No

Approximately what time is the child usually picked up from after school care?

____

Please estimate how much screen time (tablet, mobile phone, computer, TV) the child has on a typical weekday.

- ◯ None
- ◯ A maximum of 1 hour
- ◯ 1-2 hours
- ◯ 2-3 hours
- ◯ 3-4 hours
- ◯ 4-5 hours
- ◯ 5-6 hours
- ◯ More than 6 hours

Please estimate how much screen time (tablet, mobile phone, computer, TV) the child has on a typical day during the weekend.

- ◯ None
- ◯ A maximum of 1 hour
- ◯ 1-2 hours
- ◯ 2-3 hours
- ◯ 3-4 hours
- ◯ 4-5 hours
- ◯ 5-6 hours
- ◯ More than 6 hours
