# Supplementary material for: Reconstructing schoolyards with greenery to increase schoolchildren’s physical activity and mitigate climate changes in urban areas: study protocol for a stepped-wedge trial
Source: BMC Public Health. 2026 Feb 17;26:708. doi: 10.1186/s12889-026-26609-9 (PMC12930922; doi:10.1186/s12889-026-26609-9)
Supplement: Supplementary file 4 — Supplementary Material 4. [file 12889_2026_26609_MOESM4_ESM.pdf]

Perceptions of the schoolyard

Please complete the survey below.

This survey is the last one and should be answered by the child.

The child only needs to answer the questions once. If the child has already answered these questions through the same form using another guardian's email address, you may disregard this form and click 'submit' without filling in the questions.

Thank you in advance!

What do you think of the outdoor areas at your school, which you have access to during recess?

---

1) There are opportunities for many different activities
- ◯ Totally disagree
- ◯ Disagree
- ◯ Neither
- ◯ Agree
- ◯ Totally agree

2) The schoolyard is fun and challenging/exiting
- ◯ Totally disagree
- ◯ Disagree
- ◯ Neither
- ◯ Agree
- ◯ Totally agree

3) The schoolyard is good for ball games
- ◯ Totally disagree
- ◯ Disagree
- ◯ Neither
- ◯ Agree
- ◯ Totally agree

4) There is plenty of space to play in the schoolyard
- ◯ Totally disagree
- ◯ Disagree
- ◯ Neither
- ◯ Agree
- ◯ Totally agree

5) There are many different places to hang out in the schoolyard
- ◯ Totally disagree
- ◯ Disagree
- ◯ Neither
- ◯ Agree
- ◯ Totally agree

6) There is plenty of greenery, lawns, trees etc. in the schoolyard
- ◯ Totally disagree
- ◯ Disagree
- ◯ Neither
- ◯ Agree
- ◯ Totally agree

7) There are good access to unfixed equipment, e.g. balls and jump ropes in the schoolyard
- ◯ Totally disagree
- ◯ Disagree
- ◯ Neither
- ◯ Agree
- ◯ Totally agree

Overall, how do you usually feel?

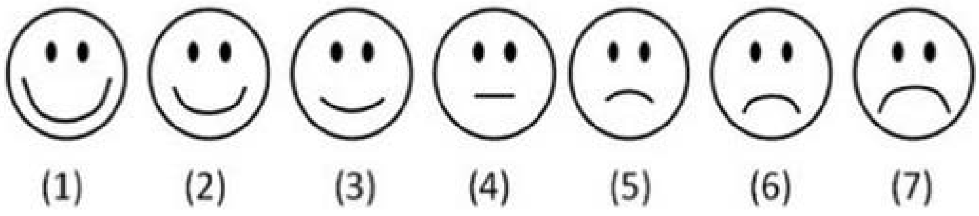

8) ◯ 1 ◯ 2 ◯ 3 ◯ 4
◯ 5 ◯ 6 ◯ 7
