# Supplementary material for: Reconstructing schoolyards with greenery to increase schoolchildren’s physical activity and mitigate climate changes in urban areas: study protocol for a stepped-wedge trial
Source: BMC Public Health. 2026 Feb 17;26:708. doi: 10.1186/s12889-026-26609-9 (PMC12930922; doi:10.1186/s12889-026-26609-9)
Supplement: Supplementary file 5 — Supplementary Material 5. [file 12889_2026_26609_MOESM5_ESM.pdf]

School policies

Please complete the survey below.

Thank you!

1) Name of school

____

2) Does your school have a health policy with the aim to work towards increasing physical activity and healthy dietary habits among the students?

- ◯ Yes, physical activity
- ◯ Yes, both physical activity and dietary habits
- ◯ Yes, dietary habits
- ◯ No neither of those
- ◯ I don't know

3) Are students allowed to use their mobile phones during breaks?

- ◯ Yes
- ◯ No
- ◯ I don't know

4) We have organized physical activities during breaks that are led by a teacher or a student.

- ◯ Every day
- ◯ 1-3 times per week
- ◯ Seldom or never

5) We have activity breaks or other types of physical activities during the lessons (apart from PE lessons)

- ◯ Every day
- ◯ 1-3 times per week
- ◯ Seldom or never
