# Supplementary material for: Reconstructing schoolyards with greenery to increase schoolchildren’s physical activity and mitigate climate changes in urban areas: study protocol for a stepped-wedge trial
Source: BMC Public Health. 2026 Feb 17;26:708. doi: 10.1186/s12889-026-26609-9 (PMC12930922; doi:10.1186/s12889-026-26609-9)
Supplement: Supplementary file 6 — Supplementary Material 6. [file 12889_2026_26609_MOESM6_ESM.pdf]

Reconstruction of Schoolyards: Effects of Schoolyard Reconstruction on Schoolchildren's Physical Activity and Health

Information for Research Participants

We would like to ask if you would like to participate in a research project. This document provides information about the project and what participation entails.

What is the project and why are we being asked to participate?

Despite growing awareness of the significant health benefits of physical activity, less than half of Swedish schoolchildren meet the current guidelines of at least 60 minutes of moderate-to-vigorous physical activity daily. The aim of this study is to evaluate how schoolyard reconstruction affects levels of physical activity and health among schoolchildren. The study is a collaborative project between The Swedish Cancer Society, Arwidssonstiftelsen, and Karolinska Institutet. The purpose of the study is to determine whether the reconstruction of schoolyards leads to increased physical activity in children, thereby promoting good health now and in the future.

Your child’s school is one of the participating schools. Through your school, we have received your contact information.

The principal investigator of the project is Karolinska Institutet, which means that the organization is responsible for the project. The project has been approved by the Swedish Ethical Review Authority, with approval number Dnr 2023-00525-01.

How will the study be conducted?

As a participating guardian, you will initially fill out a questionnaire about your child’s health. You will also answer a short questionnaire about the work and leisure activities of the guardians. Your child’s activity levels and hand strength will be measured, and these measurements will take place on the start day of the activity measurement at

your child's school. Activity levels will be measured over seven consecutive days using an accelerometer, a motion sensor worn on the child's wrist. All questionnaires are digitalized.

As a guardian, you are required to ensure that your child wears the accelerometer both day and night if possible, as this provides a basis for analyzing any correlation between sleep patterns and activity levels. The accelerometer is not waterproof and should be removed when your child showers, bathes, or swims.

Possible Consequences and Risks of Participating in the Study
There are no documented risks associated with using an accelerometer/motion sensor. Your child may find it uncomfortable to wear it at night. If that is the case, it is up to the family to decide whether to use it at night. Measuring hand strength involves no known risks.

What will happen to our information?
The project will collect and record necessary information about you and your child/children. All data in the study will be pseudonymized and presented at an aggregated level, meaning that it will not be possible to trace the results to a specific school or child. All data will be securely stored on servers at the Department of Global Public Health, Karolinska Institutet.

Your responses and your results will be handled so that no unauthorized persons can access them. All personal data will be processed in accordance with the EU General Data Protection Regulation (GDPR). Karolinska Institutet is responsible for the personal data. According to the GDPR, you have the right to access the information held about you in the project and, if necessary, have any errors corrected. You can also request that data about you be deleted, or that the processing of your personal data be restricted. However, the right to deletion and restriction of processing does not apply when the data is necessary for the ongoing research. If you wish to access the data, please contact the lead researcher Daniel Berglind at daniel.berglind@ki.se. The data protection officer can be reached at dataskyddsombud@ki.se. If you are dissatisfied with how your personal data is handled, you have the right to lodge a complaint with the Swedish Authority for Privacy Protection, which is the supervisory authority.

How will I receive information about the results of the project?
After the measurements and analysis are completed, you will receive a report with detailed data on your child’s activity patterns during the two measurement weeks. This

report can be a valuable tool in creating and/or maintaining adequate levels of physical activity for your child. Achieving the recommended levels of physical activity is linked to significant health benefits for your child, both now and in the future. The results of the study will be published in scientific journals.

Insurance and Compensation

Participants in the study are not entitled to any compensation. Children attending public schools in Stockholm are insured against accidents through the insurance company S:t Erik Försäkrings AB.

Informed Consent

Before participating in the study, you will complete a consent form where you agree to participate in the study for both yourself as a guardian and your child/children. If there are two guardians, both must consent to participation in the study. The consent form is attached to this document.

Participation is Voluntary

Your and your child's participation is voluntary, and you may withdraw from the study at any time. If you choose not to participate or wish to withdraw, you do not need to provide a reason. If you wish to withdraw, please contact the study's lead researcher.

Responsible for the Study

Karolinska Institutet is the principal investigator and data controller. The lead researcher is Daniel Berglind, who is also the contact person (details below).

Daniel Berglind | PhD | Associate Professor
Department of Global Public Health | Karolinska Institutet
Center for Epidemiology and Community Medicine
104 31 Stockholm | Solnavägen 1E
+46 70-364 47 97
Email: daniel.berglind@ki.se

Consent to Participate in the Study (Please write clearly)

To the guardians (if there are two guardians, both must sign):

I/We have received information about the study and have had the opportunity to ask questions.
I/We consent to participate in the study *"Reconstruction of Schoolyards: Effects of Schoolyard Reconstruction on Schoolchildren's Physical Activity and Health."*
I/We consent to the processing of my/our data as described in the information provided to research participants.

School of child | Class of child (Ex: 1A)

Name of child | SSN (personnummer) of child

Place and date | Signature guardian 1 | Clarification of signature

---

SSN (personnummer) guardian 1 | Email address guardian 1

Place and date | Signature guardian 2 | Clarification of signature

---

SSN (personnummer) guardian 2 | Email address guardian 2

Please provide the email address to which the report on your child's activity patterns should be sent:

Email address: ____
